# Treatment of bulky lymph nodes in locally advanced cervical cancer: boosting versus debulking

**DOI:** 10.1136/ijgc-2022-003357

**Published:** 2022-04-28

**Authors:** Ester Paulien Olthof, Hans Wenzel, Jacobus van der Velden, Anje M Spijkerboer, Ruud Bekkers, Jogchum J Beltman, Hans W Nijman, Brigitte Slangen, Ramon Smolders, Nienke van Trommel, Petra L M Zusterzeel, Ronald Zweemer, Lukas J A Stalpers, Maaike van der Aa, Constantijne Mom

**Affiliations:** 1 Department of Research and Development, Netherlands Comprehensive Cancer Organisation, Utrecht, The Netherlands; 2 Department of Gynecological Oncology, Amsterdam University Medical Centres, Centre for Gynecological Oncology Amsterdam (CGOA), Amsterdam, The Netherlands; 3 Department of Obstetrics and Gynaecology, University Medical Centre Groningen, Groningen, The Netherlands; 4 Department of Radiology, Amsterdam University Medical Centres, Amsterdam, The Netherlands; 5 Department of Obstetrics and Gynaecology, Catharina Hospital, Eindhoven, The Netherlands; 6 Department of Gynecology, Leiden University Medical Centre, Leiden, The Netherlands; 7 Department of Gynecologic Oncology, Maastricht University Medical Centre, Maastricht, The Netherlands; 8 Department of Gynecological Oncology, Erasmus Medical Centre, Rotterdam, The Netherlands; 9 Department of Gynecological Oncology, The Netherlands Cancer Institute, Antoni van Leeuwenhoek, Centre for Gynecologic Oncology Amsterdam (CGOA), Amsterdam, The Netherlands; 10 Department of Obstetrics and Gynecology, Radboud University Medical Center, Nijmegen, The Netherlands; 11 Department of Gynecological Oncology, University Medical Centre Utrecht, Utrecht, The Netherlands; 12 Department of Radiation Oncology, Amsterdam University Medical Centres, Amsterdam, The Netherlands

**Keywords:** Cervical Cancer, Lymphatic Metastasis, Radiotherapy, Surgical Oncology, Postoperative complications

## Abstract

**Objective:**

Treatment strategies for bulky lymph nodes in patients with locally advanced cervical cancer scheduled for definitive chemoradiation include nodal boosting with radiotherapy, surgical debulking, or both. The aim of this retrospective cohort study was to compare survival and toxicity in patients receiving these treatments and to compare them with a group that received neither form of treatment.

**Methods:**

Women diagnosed between January 2009 and January 2017 with International Federation of Gynecology and Obstetrics (FIGO) 2009 stage IB2, IIA2–IVA cervical cancer with lymph nodes ≥1.5 cm without upper limit on pretreatment imaging and treated with definitive chemoradiation were selected from the Netherlands Cancer Registry. Patients were categorized by intention-to-treat strategy: boosting, debulking, or neither treatment, with subgroup analysis for patients receiving both treatments, that is, debulking with boosting. Overall and relapse-free survival outcomes were compared by Kaplan-Meier and Cox regression analyses and toxicity by logistic regression analysis.

**Results:**

Of 190 patients, 101 (53%) received only nodal boosting, 31 (16%) debulking alone, 29 (15%) debulking combined with boosting, and 29 (15%) received neither treatment. The 5 year overall and relapse-free survival for the treatment groups were 58%, 45% and 45% (p=0.19), and 47%, 44% and 46% (p=0.87), respectively. Multivariable Cox regression analyses demonstrated no differences in overall and relapse-free survival. Combination of debulking with boosting was associated with decreased overall and relapse-free survival compared with debulking alone (HR 2.47, 95% CI 1.22 to 5.00; and HR 2.37, 95% CI 1.14 to 4.93). Nodal boosting was independently associated with a decreased toxicity risk compared with debulking strategy (OR 0.37, 95% CI 0.16 to 0.83).

**Conclusions:**

This study showed no survival benefit from either nodal boosting or debulking strategy in patients with suspicious bulky nodes. Nodal boosting might, however, be associated with less toxicity. Dual treatment with debulking and boosting showed a worse survival outcome because this group probably represents patients with poor prognostic factors.

**HIGHLIGHTS**

- Treatment of bulky nodes (≥1.5 cm) by boosting or debulking in patients with advanced cervical cancer showed similar survival rates.
- Nodal boosting strategy might be associated with less toxicity.
- Poor survival after debulking with boosting might be related to this group’s poor prognostic factors.

## Introduction

The age-standardized incidence rate of cervical cancer was 5.2 per 100 000 women for developed countries in 2020.^1^ Of these women, approximately 40% were diagnosed with locally advanced disease, defined as International Federation of Gynecology and Obstetrics (FIGO) 2009 stage IB2, IIA2–IVA, with a 5 year relative survival rate of ~58%.^2 3^ Survival is worse in patients with lymph node metastases and depends on the number, size, and affected region of nodal metastases.^4 5^ Macroscopically enlarged nodes are also known as ‘bulky’ nodes and can be defined as nodes with a short axis of ≥1.5 or ≥2.0 cm on imaging, but an unambiguous definition is lacking.^6–10^ For bulky nodes, standard dose of conventional external beam radiation (50–60 Gray (Gy)) may be insufficient for sterilization, and additional treatment may be warranted.^4 7–9 11–13^

Currently, two main strategies are used to treat bulky nodes: high-dose boost irradiation as part of standard chemoradiation and nodal debulking prior to definitive chemoradiation.^14^ Debulking nodal tumor load might increase the chance of complete sterilization by chemoradiation and decrease the risk of toxicity by avoiding a boost. To date, there has been little agreement on the most effective and safe strategy, with only a few studies evaluating the impact on survival. Some studies demonstrated effective nodal control by boosting in patients with suspicious nodes on imaging,^10 13 15–17^ while others showed improved survival after nodal debulking.^6 18–20^ Furthermore, both strategies are associated with different toxicities: surgical complications versus genitourinary and gastrointestinal toxicity.^6 13 21^ Unfortunately, direct comparative studies on survival or toxicity are missing.

This retrospective study aims to compare intention-to-treat strategies for bulky node(s)—boosting, debulking, or neither treatment—as part of a chemoradiation treatment plan in patients with locally advanced cervical cancer and suspicious bulky nodes on imaging. Relapse-free survival, overall survival, and toxicity were compared among groups.

## Methods

### Study Design

With Privacy Review Board approval (No 210029) of the Netherlands Cancer Registry, we performed a nationwide retrospective cohort study analyzing data between January 2009 and January 2017 from the Registry, which contains data of >95% of all patients with cancer in the Netherlands. The following inclusion criteria were used: (1) FIGO 2009 stage IB2, IIA2–IVA, (2) suspicious or inconclusive pelvic and/or para-aortic bulky nodes on imaging (CT, positron emission tomography (PET)-CT, MRI, or PET-MRI), and (3) treatment with curative intent (radiotherapy alone, combined with chemotherapy, or hyperthermia). Patients with neuroendocrine carcinoma or treatment with neoadjuvant chemotherapy were excluded. Details of chemoradiation at a patient level are not available, but we assumed that patients were treated according to the Dutch guidelines: external beam radiation (total dose 45–50 Gy) with concurrent single-agent chemotherapy (cisplatin weekly 40 mg/m²), and brachytherapy until a minimal dose equivalent of 80 Gy.^22^ Extended-field radiotherapy was indicated if common iliac or para-aortic regions were involved, following the EMBRACE protocol.^23^ Although there is no clear definition, based on previous studies, we defined bulky nodes as ≥1.5 cm short axis without upper limit, with subgroup analysis for those ≥2.0 cm.^6 7 10^ All patients were categorized according to intention-to-treat strategy for bulky nodes: a ‘boosting’ only, ‘debulking’, or ‘neither’ group, the latter for patients without additional nodal treatment. Patients who were treated with debulking but also received boosting were allocated to the debulking group, as allocation was based on an intention-to-treat strategy.

Nodal characteristics (including short-axis diameter and radiological judgment) were registered for five regions: pelvic left/right, common iliac left/right, and para-aortic. Data on patient, tumor, and treatment characteristics were collected. Postoperative complications were noted for those who had surgery and defined as any complication ≤30 days from surgery, scored as grade ≥2 on the Clavien-Dindo scale.^24^ Radiotherapy and chemotherapy related toxicities were defined as any complication ≤6 months after starting treatment, classified as grade ≥3 of the Common Terminology Criteria for Adverse Events (CTCAE), version 4.0.^25^ In accordance with the journal’s guidelines, we will provide our data for the reproducibility of this study in other centers if requested.

### Statistical Analysis

Continuous variables were compared by the Mann-Whitney U test or Kruskal-Wallis test, while discrete variables were assessed using the Fisher exact test. The primary outcomes, relapse-free and overall survival, were defined as the interval from the start of primary therapy to the date of recurrence and from diagnosis to death, respectively. The date of death was obtained by annual linkage with the Personal Records Database. Survival analyses were performed using the Kaplan-Meier method and the log-rank test. Furthermore, Cox regression analyses were used for calculating HRs, with 95% CIs. Subgroup analyses were performed for patients who underwent surgical debulking with and without additional boosting to account for heterogenicity within this treatment group. The multivariable models for the subgroup analyses included fewer confounders to avoid model overfitting in the case of fewer observations. Logistic regression analysis was used to calculate ORs with 95% CIs for toxicity. A p value<0.05 was considered significant, and South Texas Art Therapy Association SE 17 (StataCorp, College Station, TX) software was used for all analyses.

## Results

### Patient and Treatment Characteristics

In this study, 190 patients with bulky nodes were included (Online supplemental figure 1), of which 53% received nodal boosting (n=101), 32% surgical debulking (n=60), and 15% neither treatment (n=29). The suspicious bulky nodes in patients who received debulking were larger (median 22 mm) than the nodes of patients treated by boosting (18 mm) or without additional nodal treatment (17 mm; p<0.001), and most (≥79%) were located in the pelvic region (Table 1). Compared with the boosting group, the median interval between diagnosis and chemoradiation was 14 and 7 days longer in the debulking and neither group, respectively. Primary treatment differed between the groups. The group without debulking or boosting received the least comprehensive treatment, with less chemotherapy and/or hyperthermia (p<0.001), brachytherapy (p<0.001), and extended field radiotherapy (p=0.002). Out of the debulking procedures, 47% were performed with a combination of pelvic and/or para-aortic lymphadenectomy, and the majority (67%) were performed by open surgery. Histological examination of bulky nodes was negative in four patients (7%) after surgical resection and was only performed in the debulking group. The median number of retrieved nodes was nine (range 1–33), with a median of three positive nodes (range 0–22).

10.1136/ijgc-2022-003357.supp1 Supplementary data

**Table 1:** Patient characteristics, categorized per treatment group for patients with bulky nodes ≥1.5 cm

Characteristics	Overall (n=190)	Boosting (n=101)	Debulking (n=60)	Neither (n=29)	p-value
Age (years)	51 (25–92)	51 (27–92)	50 (25–77)	55 (31–83)	0.005*
Charlson morbidity index					0.56
0	121 (64%)	63 (62%)	40 (67%)	18 (62%)	
1	23 (12%)	14 (14%)	5 (8%)	4 (14%)	
≥2	13 (7%)	9 (9%)	4 (7%)	–	
Unknown	33 (17%)	15 (15%)	11 (18%)	7 (24%)	
Smoking (yes)	62 (33%)	33 (33%)	18 (30%)	11 (38%)	0.75
Pretreatment SCC-Ag† (ng/mL)	10.9 (0.1–278.0)	8.0 (0.1–224.3)	16.4 (1.0–176.0)	11.9 (0.3–278.0)	0.14
FIGO 2009 stage					0.16
IB2	30 (16%)	15 (15%)	14 (23%)	1 (3%)	
II	105 (55%)	54 (53%)	33 (55%)	18 (62%)	
III	46 (24%)	28 (28%)	11 (18%)	7 (24%)	
IVA	9 (5%)	4 (4%)	2 (3%)	3 (10%)	
Primary tumor size					0.08
≤4	31 (16%)	13 (13%)	11 (18%)	7 (24%)	
>4	156 (82%)	87 (86%)	49 (82%)	20 (69%)	
Unknown	3 (2%)	1 (1%)	0 (0%)	2 (7%)	
Histological type					0.92
Squamous	165 (87%)	87 (86%)	53 (88%)	25 (86%)	
Non-squamous	25 (13%)	14 (14%)	7 (12%)	4 (14%)	
Bulky node size (mm)	19 (15–86)	18 (15–86)	22 (15–83)	17 (15–60)	<0.001*
Region of bulky node‡					0.21
Pelvic	158 (83%)	84 (83%)	51 (85%)	23 (79%)	
Common iliac	12 (6%)	7 (7%)	5 (8%)	–	
Para-aortic	20 (11%)	10 (10%)	4 (7%)	6 (21%)	
Diagnosis to primary treatment interval (days)	53 (11–128)	48 (11–96)	62 (28–128)	55 (30–125)	<0.001*
Primary treatment					<0.001*
CRT	149 (78%)	75 (74%)	57 (95%)	17 (59%)	
(C)HRT	25 (13%)	17 (17%)	1 (2%)	7 (24%)	
RT only	16 (8%)	9 (9%)	2 (3%)	5 (17%)	
Brachytherapy (yes)	178 (94%)	98 (97%)	58 (97%)	22 (76%)	<0.001*
Nodal boost (yes)	130 (68%)	101 (100%)	29 (48%)	–	<0.001*
Radiotherapy field					0.002*
Pelvic	114 (60%)	64 (63%)	28 (47%)	22 (76%)	
Pelvic +para-aortic	71 (37%)	37 (37%)	27 (48%)	5 (17%)	
Unknown	5 (3%)	–	3 (5%)	2 (7%)	
Follow-up (months)	45 (3–144)	49 (3–143)	42 (5–144)	40 (8–134)	0.85
Recurrence	93 (49%)	49 (49%)	31 (52%)	13 (45%)	0.83
Recurrence location§					0.31
Central pelvic	14 (15%)	7 (14%)	4 (13%)	3 (23%)	0.62
Lateral pelvic	24 (26%)	11 (22%)	10 (32%)	3 (23%)	0.64
Para-aortic	30 (32%)	16 (33%)	11 (35%)	3 (23%)	0.75
Distant	70 (75%)	36 (73%)	24 (77%)	10 (77%)	0.94
Unknown	1 (1%)	1 (2%)	–	–	–
Vital status					0.26
Alive	86 (45%)	51 (51%)	25 (42%)	10 (34%)	
Deaths	104 (55%)	50 (49%)	35 (58%)	19 (66%)	

Data are the number of patients (percentage) or median (range).

*Statistically significant.

†For squamous cell type only.

‡Most cranial lymph node region was decisive.

§Some patients had multiple recurrence locations.

SCC-Ag, squamous cell antigen; FIGO, International Federation of Gynecology and Obstetrics; CRT, chemoradiation; (C)HRT, (chemotherapy with) hyperthermia and radiotherapy; RT, radiotherapy.

### Oncological Outcome

**Figure 1:**
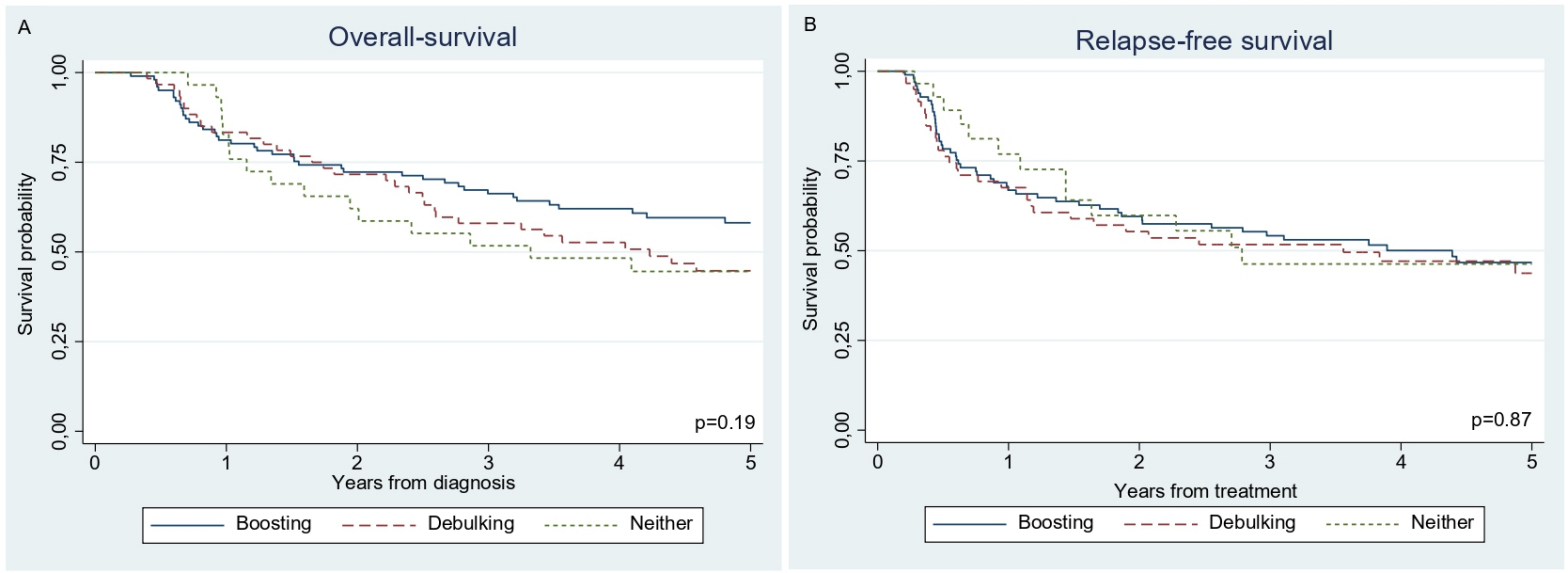
Kaplan-Meier estimates after treatment of women with locally advanced cervical cancer plus bulky nodes (≥1.5 cm). (A) Overall survival; (B) relapse-free survival.

With a median follow-up of 45 months (range 3–144), 93 recurrences (49%) and 104 deaths (55%) were observed (Table 1). Infield recurrences were observed in 34 (36.5%) of 93 patients with a relapse, and distant relapse was the most common cause of recurrence and death (≥73%). The 5 year overall survival was 58% (95% CI 48% to 67%) in the boosting, 45% (32%–57%) in the debulking, and 45% (26%–61%) in the neither treatment group (p=0.19; Figure 1A). Additionally, there were no differences observed in the 5 year relapse-free survival (Figure 1B) among the treatment groups, which was 47% (36%–57%) after boosting, 44% (30%–57%) after debulking, and 46% (26%–65%) after no treatment (p=0.87). Results of multivariable analyses are presented in Table 2. Overall and relapse-free survival were not affected by the different treatment strategies.

**Table 2:** Multivariable analysis regarding overall and relapse-free survival

Variables	Overall survival			Relapse-free survival		
	HR	95% CI	p-value	HR	95% CI	p-value
Treatment group						
Debulking	1.00	Reference		1.00	Reference	
Boosting	0.65	0.51 to 1.03	0.07	0.85	0.53 to 1.37	0.51
Neither	0.85	0.45 to 1.59	0.61	0.78	0.38 to 1.61	0.51
Age†	1.02	1.01 to 1.03	0.007*	1.01	0.99 to 1.02	0.42
Bulky node location						
Pelvic	1.00	Reference		1.00	Reference	
Common iliac	1.20	0.57 to 2.51	0.64	1.00	0.44 to 2.29	1.00
Para-aortic	1.42	0.79 to 2.57	0.25	1.37	0.70 to 2.70	0.36
Bulky node size†	1.00	0.98 to 1.02	0.97	1.01	0.99 to 1.03	0.36
FIGO 2009 stage						
IB2	1.00	Reference		1.00	Reference	
II	1.03	0.54 to 1.97	0.94	1.00	0.53 to 1.90	1.00
III	1.50	0.73 to 3.08	0.27	1.36	0.66 to 2.80	0.40
IVA	2.82	1.04 to 7.66	0.042*	2.07	0.69 to 6.25	0.20
Primary tumor size (cm)						
≤4	1.00	Reference		1.00	Reference	
>4	1.60	0.90 to 2.86	0.11	1.44	0.77 to 2.70	0.25

*Statistically significant.

†Continuos scale.

FIGO, International Federation of Gynecology and Obstetrics.;

### Toxicity

Toxicities related to surgery, radiotherapy, and chemotherapy are presented in Table 3. By definition, postoperative complications only occurred in the debulking group (10%), and infection was most common (7%; n=4). The number of patients experiencing radiotherapy-related (p=0.29), chemotherapy-related (p=0.16), or any toxicity (p=0.06) did not differ between treatment groups. Additionally, toxicity in the debulking group did not differ between patients with and without a lymphadenectomy (29% and 41%; p=0.42). After adjusting for age, primary treatment, extended-field radiotherapy, and bulky node size, nodal boosting was associated with less toxicity (OR 0.37; 95% CI 0.16 to 0.83) compared with debulking (Online supplemental table 1).

10.1136/ijgc-2022-003357.supp2 Supplementary data

**Table 3:** Toxicities related to surgery (grade ≥2), radiotherapy, and chemotherapy (grade ≥3) per treatment group

	Boosting (n=101)	Debulking (n=60)	Neither (n=29)	p-value
Surgery				
Intraoperative injury	–	2 (3%)	–	
Infection	–	4 (7%)	–	
IC-admission	–	1 (2%)	–	
Blood transfusion	–	1 (2%)	–	
Total†	–	8	–	
Total patients	–	6 (10%)	–	
Radiotherapy				
Urological	3 (3%)	–	2 (7%)	0.10
Gastrointestinal	9 (9%)	5 (8%)	6 (21%)	0.19
Genital	1 (1%)	–	1 (4%)	0.38
Other	2 (2%)	2 (3%)	2 (7%)	0.30
Total†	15	5	2	
Total patients	15 (15%)	7 (12%)	7 (24%)	0.29
Chemotherapy				
Nausea/vomiting	3 (3%)	3 (5%)	–	0.62
Nephrotoxicity	4 (4%)	1 (2%)	–	0.57
Ototoxicity	1 (1%)	–	–	
Bone marrow depression	–	4 (7%)	1 (3%)	0.022*
Malaise/fatigue	–	2 (3%)	1 (3%)	0.14
Neurotoxicity	1 (1%)	–	–	
Other	3 (3%)	3 (5%)	3 (10%)	0.19
Total†	12	4	9	
Total patients	9 (9%)	11 (18%)	5 (17%)	0.16
Total adverse events				
Total†	27	17	11	
Total patients	21 (21%)	21 (35%)	11 (38%)	0.06

*Statistically significant.

†Some patients experienced multiple toxicities.

IC, intensive care.;

### Subgroup Analyses

**Figure 2:**
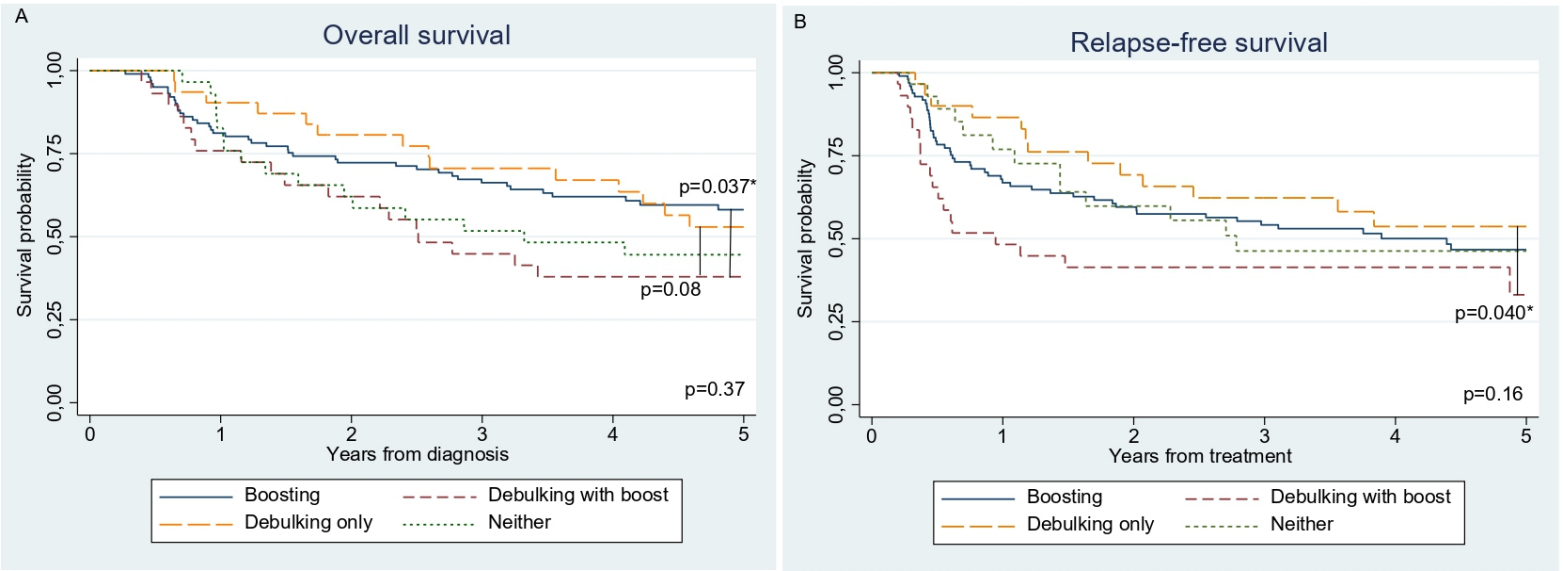
Kaplan-Meier estimates after treatment of women with locally advanced cervical cancer plus bulky nodes (≥1.5 cm), with subgroup analyses for nodal debulking with and without boost. (A) Overall survival; (B) relapse-free survival.

Subgroup analysis of patients who received debulking with (n=29) or without (n=31) boosting demonstrated a worse 5 year relapse-free and overall survival for those who had boosting (33%, 95% CI 15% to 53%; and 38%, 95% CI 21% to 55%), while the survival of those without boosting was comparable to boosting alone (54%, 95% CI 34% to 70%; and 53%, 95% CI 34% to 69%) (Figure 2A, B). In multivariable analysis, nodal debulking with boost was negatively associated with overall (HR 2.47, 95% CI 1.22 to 5.00) and relapse-free survival (HR 2.37, 95% CI 1.14 to 4.93), compared with debulking alone (Online supplemental table 2). Toxicity did not differ between the boosted and non-boosted groups (n=10, 48% vs n=11, 52%).

Subgroup analysis for bulky nodes ≥2 cm included 35, 48, and 9 patients in the boosting, debulking, and neither treatment groups, respectively. Although patients and treatment characteristics were more balanced among the treatment groups (Online supplemental table 3), the 5 year overall (53%, 46%, 53%; p=0.83) and relapse-free survival (43%, 43%, 36%; p=0.91) did not differ between the boosting, debulking, and neither treatment groups. Furthermore, overall and relapse-free survival were not affected by the different treatment strategies in multivariable analysis (Online supplemental table 2). No differences were observed regarding toxicities (data not shown).

## Discussion

### Summary of Main Results

In this study, we were unable to demonstrate superiority of any one of three treatment strategies on overall or relapse-free survival. However, boosting alone might be associated with less toxicity compared with the debulking strategy, with or without boosting. Subgroup analysis for bulky nodes ≥2 cm demonstrated similar survival results among the treatment groups, although the sample size is too small to draw firm conclusions. Subgroup analysis for debulking with or without boosting demonstrated that dual treatment by debulking with boosting was independently associated with a worse survival outcome compared with debulking alone. This is most likely related to the selection of eligible patients for dual treatment, a subgroup with poor prognostic factors.

### Results in the Context of Published Literature

This study directly compared different treatment strategies in one patient cohort with cervical cancer and suspicious bulky nodes. In literature, the few studies on this topic focus either on nodal debulking or boosting. Three studies on debulking demonstrated survival benefits, but were performed before concurrent chemoradiation was standard care for locally advanced cervical cancer.^18–20^ These studies showed that patients in whom microscopic and macroscopic nodal metastases were removed during surgical staging had comparable 5 year relapse-free survival rates (50%–57% versus 43%–57%, respectively), while patients with unresectable nodes had a survival of 0%. Vascular and nervous adherence or invasion was the main cause of unsuccessful resections, and none of the nodes were boosted. More recently, another study on nodal debulking demonstrated no survival benefits in patients with locally advanced cervical cancer.^26^ Laparoscopic para-aortic staging was combined with or without debulking suspicious pelvic nodes on imaging. Patients with suspicious or histologically confirmed pelvic metastases received nodal boosting in addition to chemoradiation. The 5 year disease-free (both ~55%) and overall survival did not differ between the debulked (~65%; n=164) and non-debulked groups (63%; n=111). Notably, the suspicious nodes on MRI or PET-CT were relatively small (range 1.0–1.8 cm), and only 43% of the debulked nodes were positive on pathological examination.

Most recent studies on strategies for bulky nodes focus on boosting in relatively small patient cohorts.^10 13 15–17^ Bulky nodes on imaging are associated with lower loco-regional control rates, which could be increased by radiotherapy dose escalation (>55.8 Gy) in patients with stage IB–IVA cervical cancer treated with definitive chemoradiation.^13 17^ Some studies achieve local control in 83%–92% of patients after nodal boosting of suspicious pelvic and/or para-aortic nodes, with disease-free and overall survival rates of 73%–76% and 58%–71%, respectively.^15 16^ In a study on patients with locally advanced cervical cancer and suspicious pelvic nodes treated by definitive chemoradiation with (n=36) and without (n=31) nodal boosting, the 5 year recurrence-free (49% vs 65%; p=0.17) and overall survival (74% vs 81%; p=0.14) did not differ between groups, which is in line with our results.^27^ Notably, the survival rates of these studies on boosting are considerably higher than ours, which might be related to varying definitions of bulky nodes (≥1.0 to ≥2.4) and boost administration (50.4–63.0 Gy). Remarkably, none of the suspicious nodes in the above-listed studies were histologically confirmed. Therefore, it is difficult to compare separate studies on nodal boosting or debulking.

Pelvic and para-aortic nodes are adjacent to high-risk organs for radiotherapy. Dose escalation could therefore lead to increased toxicity. The potential benefit from debulking nodal tumor load in terms of toxicity could not be demonstrated in our study because nodal boosting was associated with less toxicity. This can be explained by the contribution of surgery-related toxicities, which naturally can only occur after debulking. Even though open surgery was the most common approach in our study, the 10% of surgery-related complications is in concordance with toxicity described in the literature on surgical staging in locally advanced cervical cancer, including two studies with a laparoscopic approach.^20 28 29^ Studies on nodal boosting report higher acute (4%–41%) and late (4%–29%) radiotherapy-related toxicity (grade ≥2) compared with our cohort (15%–17%).^10 15 16 27^ This could be attributed to the shorter follow-up (≤6 months), unreported toxicity in patient records, or potentially other doses of boost irradiation in our study.

Overall, studies on treatment strategies for bulky nodes in cervical cancer are scarce, and direct comparisons of nodal boosting with debulking are lacking. It is important to keep in mind that the nodes in most studies were generally <1.5 cm and that the studies on nodal boosting might have included false positives, which could positively affect survival rates.^10 13 15–17^ Therefore, there is a need to directly compare both strategies within one cohort.

### Strengths and Weaknesses

Our study is based on national data of a relatively large retrospective study cohort, allowing correction for several confounders. It provides data from real-world clinical practice but is unfortunately also inherently associated with the risk of bias. First, histological confirmation of suspicious bulky nodes was only performed after debulking, while the positive predictive value for nodal imaging is only 55%–96%.^30^ Therefore, both ‘boosting’ and ‘neither’ groups probably contain false-positive bulky nodes. We have analyzed a subgroup with nodes ≥2.0 cm, in which positive predictive values were likely higher. However, this has led to small cohort sizes limiting statistical power. Second, the ‘neither’ group probably represents a poor prognostic group with higher age and lower performance scores, as primary treatment in this group was less comprehensive. Also, extended-field radiotherapy was more commonly applied after debulking than in the boosting group, which might reflect those with a poorer prognosis. However, extended-field radiotherapy was equally common in the treatment groups with bulky nodes ≥2 cm, with similar survival outcomes as in the whole patient group. Another limitation is the lack of details on chemoradiation modalities and boost irradiation, including dose and location. This is especially important in the debulking with boosting group because the boost might also have targeted other suspicious nodes and not only the location of the (possibly incompletely) resected node. Lastly, the debulking procedures in our study were extensive, with nine median retrieved nodes (range 1–33) and a combination with lymphadenectomy in 47%. More extensive procedures are associated with higher toxicity, which might be reflected by our results on toxicity after debulking. Despite these limitations, this study represents the largest cohort of patients comparing different treatment strategies of bulky nodes in locally advanced cervical cancer and adds valuable information to existing literature.

### Implications for Practice and Future Research

A randomized clinical trial on strategies for bulky nodes might overcome bias related to retrospective study designs. However, the feasibility might be poor due to insufficient eligible patients, and international collaboration would be necessary. However, as ≥73% of the recurrences included distant metastases, strategies that may reduce distant relapse rather than achieving local control by boosting or debulking may be more urgently warranted for this patient group.

## Conclusion

In conclusion, we were unable to demonstrate superiority of the addition of nodal boosting or debulking over chemoradiation on overall and relapse-free survival in patients with locally advanced cervical cancer and suspicious bulky nodes of ≥1.5 cm on imaging. Furthermore, reducing tumor load by nodal debulking might increase the risk of toxicity compared with nodal boosting. However, these results must be interpreted cautiously because of our retrospective study design. Finally, the combination of debulking with boosting was associated with decreased survival outcomes, but this group probably represents patients with poor prognostic factors. As none of the strategies were superior to survival, shared decision-making and individualized treatment seem to be the best approach for patients with bulky nodes.

## Data Availability

Data are available upon reasonable request.
