# Supplementary figures and images for: Treatment of bulky lymph nodes in locally advanced cervical cancer: boosting versus debulking
Source: Int J Gynecol Cancer. 2022 Apr 28;32(7):861–8. doi: 10.1136/ijgc-2022-003357 (PMC9279830; doi:10.1136/ijgc-2022-003357)

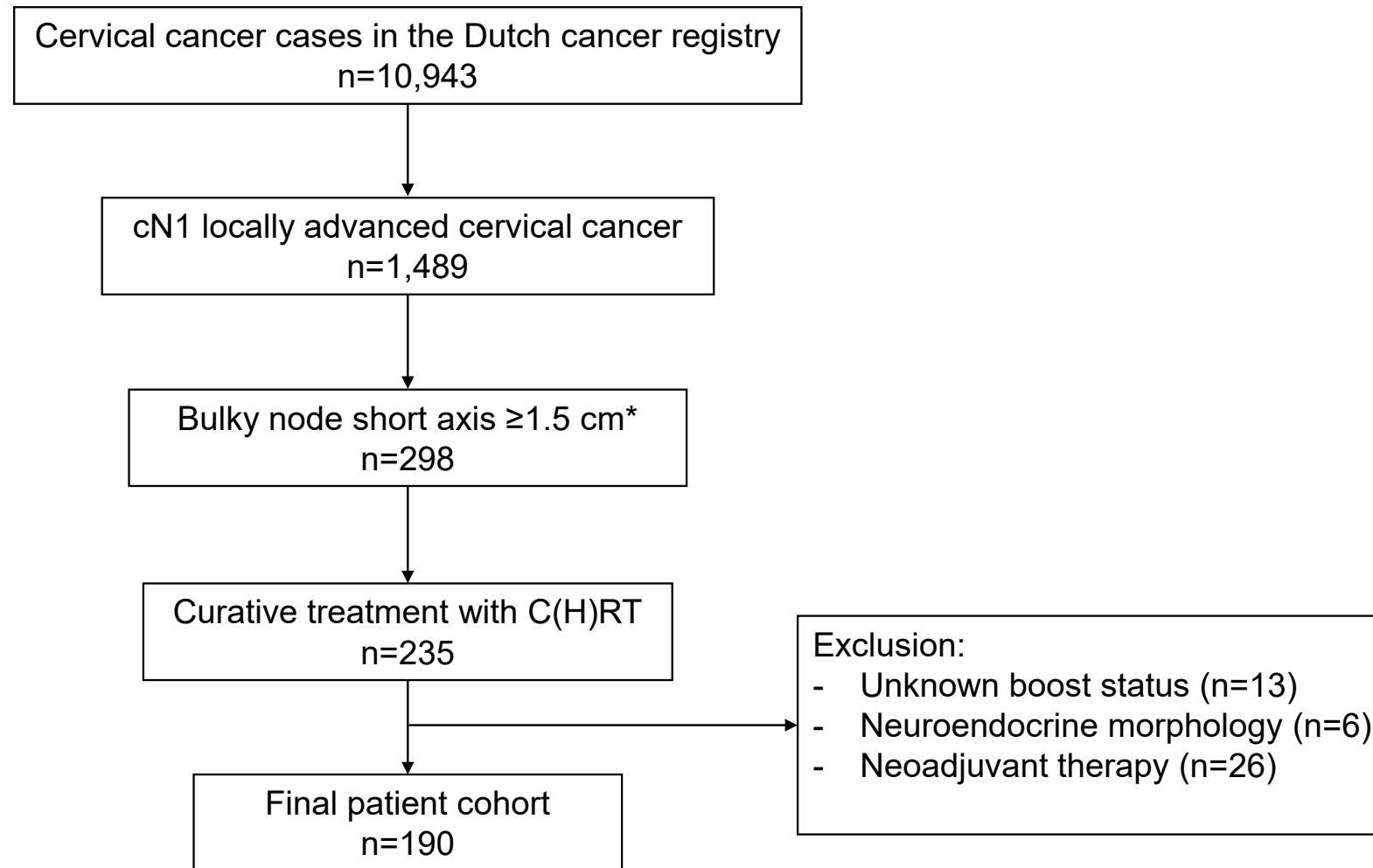

Supplement: Supplementary data [file ijgc-2022-003357supp001.pdf]
