# Supplementary material for: Treatment of bulky lymph nodes in locally advanced cervical cancer: boosting versus debulking
Source: Int J Gynecol Cancer. 2022 Apr 28;32(7):861–8. doi: 10.1136/ijgc-2022-003357 (PMC9279830; doi:10.1136/ijgc-2022-003357)
Supplement: Supplementary data [file ijgc-2022-003357supp002.pdf]

SUPPLEMENTAL MATERIAL

Figure S1 Flow chart of patient inclusion with locally advanced cervical cancer and suspicious bulky nodes ≥1.5 cm.

Abbreviations: cN1, presence of regional lymph node metastasis bases on clinical examination and imaging; C(H)RT, radiotherapy with or without chemotherapy and/or hyperthermia. * Data were collected on project basis for cervical cancer patients diagnosed between 2009 and 2017.

Table S1 Logistic regression analysis for total patients experiencing surgery, radiotherapy or chemotherapy related toxicities.

Variables	OR	95% CI	p-value
**Therapy group**			
Debulking	1.00	Reference	
Boosting	0.37	0.16-0.83	0.017*
Neither	0.89	0.30-2.70	0.84
**Age†**	1.04	1.01-1.07	0.003*
**Primary treatment**			
CRT	1.00	Reference	
(C)HRT	1.17	0.42-3.24	0.23
RT only	0.06	0.01-0.59	0.015*
**Radiotherapy field**			
Pelvic	1.00	Reference	
Pelvic + para-aortic	1.20	0.58-2.47	0.62
**Bulky node size†**	1.03	0.99-1.07	0.18
**Constant**	0.04	0.01-0.23	<0.001*

Abbreviations: RT, radiotherapy; CRT, chemoradiation; (C)HRT, (chemotherapy with) hyperthermia and radiotherapy; OR, odds ratio; CI, confidence interval. † Continues scale; * statistically significant.

Olthof EP, *et al. Int J Gynecol Cancer* 2022; 32:861–868. doi: 10.1136/ijgc-2022-003357

Table S2 Multivariable subgroup analysis for nodal debulking with and without boost, and for bulky nodes ≥2 cm regarding overall and relapse-free survival.

	Debulking with/without boost (n=60)						Bulky nodes ≥2 cm (n=92)					
	Overall survival			Relapse-free survival			Overall survival			Relapse-free survival		
**Variables**	**HR**	**95% CI**	**p-value**	**HR**	**95% CI**	**p-value**	**HR**	**95% CI**	**p-value**	**HR**	**95% CI**	**p-value**
**Therapy group**												
Debulking only	1.00	Reference		1.00	Reference		1.00	Reference		1.00	Reference	
Debulking with boost	2.47	1.22-5.00	0.012*	2.37	1.14-4.93	0.021*						
Boosting							0.67	0.35-1.28	0.23	0.90	0.48-1.69	0.75
Neither							0.61	0.23-1.65	0.33	0.73	0.25-2.13	0.57
**Age†**	1.03	1.00-1.06	0.07	1.02	0.99-1.05	0.12	1.02	1.00-1.04	0.023*	1.02	1.00-1.04	0.08
**Bulky node location**												
Pelvic	1.00	Reference		1.00	Reference		1.00	Reference		1.00	Reference	
Common iliac	0.80	0.24-2.69	0.72	0.57	0.13-2.42	0.45	1.08	0.33-3.54	0.90	0.37	0.05-2.71	0.33
Para-aortic	3.10	1.04-9.23	0.042*	2.16	0.64-7.29	0.22	1.70	0.69-4.18	0.25	1.92	0.74-4.99	0.18
**Bulky nodes size†**	1.01	0.98-1.04	0.49	1.02	0.99-1.05	0.29	1.01	0.98-1.03	0.57	1.01	0.98-1.03	0.59

Abbreviations: HR, hazard ratio; CI, confidence interval. † Continues scale; * statistically significant.

Table S3 Patients and treatment characteristics categorized per treatment group for patients with bulky nodes ≥2 cm.

Characteristics	Overall (n=92)	Boosting (n=35)	Debulking (n=48)	Neither (n=9)	P-value
**Age** (years)	53 (27-86)	50 (25-77)	54 (31-82)	52 (25-77)	0.07
**Charlson comorbidity index**					0.19
0	55 (60%)	20 (57%)	21 (67%)	16 (62%)	
1	14 (15%)	7 (20%)	5 (10%)	2 (8%)	
≥2	7 (8%)	4 (11%)	3 (6%)	3 (12%)	
Unknown	16 (17%	4 (11%)	8 (17%)	5 (19%)	
**Squamous cell antigen†** (ng/mL)	11.4 (0.3-224.3)	7.5 (0.5-224.3)	14.8 (1.0-176.0)	5.0 (0.3-79.5)	0.24
**FIGO 2009 stage**					0.21
IB2	17 (18%)	4 (11%)	13 (27%)	-	
II	48 (52%)	18 (51%)	22 (46%)	8 (89%)	
III	23 (25%)	11 (31%)	11 (23%)	1 (11%)	
IVA	4 (4%)	2 (6%)	2 (4%)	-	
**Primary tumor size** (cm)					0.42
≤4	16 (18%)	4 (11%)	11 (23%)	1 (13%)	
>4	75 (82%)	31 (89%)	37 (77%)	7 (88%)	
**Histology**					>0.99
Squamous	82 (89%)	31 (89%)	43 (90%)	8 (89%)	
Non-squamous	10 (11%)	4 (11%)	5 (10%)	1 (11%)	
**Bulky node size** (mm)	25 (20-86)	24 (20-86)	24 (20-83)	26 (20-60)	0.64
**Bulky node location‡**					0.64
Pelvic	81 (88%)	29 (83%)	44 (92%)	8 (89%)	
Common iliac	4 (4%)	2 (6%)	2 (4%)	-	
Para-aortic	7 (8%)	4 (11%)	2 (4%)	1 (11%)	
**Diagnosis to primary treatment interval** (days)	55 (11-125)	50 (11-71)	62 (28-107)	61 (34-125)	<0.001
**Primary treatment**					0.005*
CRT	75 (82%)	24 (69%)	45 (94%)	6 (67%)	
(C)HRT	10 (11%)	8 (23%)	1 (2%)	1 (11%)	
RT only	7 (8%)	3 (9%)	2 (4%)	2 (22%)	
**Brachytherapy** (yes)	85 (92%)	33 (94%)	46 (96%)	6 (67%)	0.023*
**Nodal boost** (yes)	57 (62%)	35 (100%)	22 (46%)	-	<0.001*
**Radiotherapy field**					0.09
Pelvic	48 (52%)	19 (54%)	22 (46%)	7 (78%)	
Pelvic + para-aortic	40 (43%)	16 (46%)	23 (48%)	1 (11%)	
Other/unknown	4 (4%)	-	3 (6%)	1 (11%)	
**Recurrence**	48 (52%)	19 (54%)	25 (52%)	4 (44%)	0.91
**Recurrence location§**					0.75
Central pelvic	6 (13%)	3 (16%)	3 (12%)	-	>0.99

BMJ Publishing Group Limited (BMJ) disclaims all liability and responsibility arising from any reliance placed on this supplemental material which has been supplied by the author(s)

Lateral pelvic	10 (21%)	4 (21%)	6 (24%)	-	0.87
Para-aortic	17 (35%)	5 (26%)	11 (44%)	1 (25%)	0.48
Distant	37 (77%)	14 (74%)	20 (80%)	3 (75%)	0.88
Unknown	1 (2%)	1 (5%)	-	-	-
**Vital status**					0.83
Alive	43 (47%)	18 (51%)	21 (44%)	4 (44%)	
Deaths	49 (53%)	17 (49%)	27 (56%)	5 (56%)	

Abbreviations: FIGO, International Federation of Gynecology and Obstetrics; CRT, chemoradiation; (C)HRT, (chemotherapy with) hyperthermia and radiotherapy; RT, radiotherapy. † Pretreatment level for squamous cell type only; ‡ most cranial lymph node region was decisive; § some patients had multiple recurrence locations; * statistically significant.

Olthof EP, *et al. Int J Gynecol Cancer* 2022; 32:861–868. doi: 10.1136/ijgc-2022-003357
